# Supplementary material for: Artificial intelligence supported anemia control system (AISACS) to prevent anemia in maintenance hemodialysis patients
Source: Int J Med Sci. 2021 Feb 22;18(8):1831–9. doi: 10.7150/ijms.53298 (PMC7976591; doi:10.7150/ijms.53298)
Supplement: Supplementary file 1 — Supplementary tables. [file ijmsv18p1831s1.pdf]

Supplementary table 1. Background

	Hospital S		Hospital K
	S_1	S_2	K_1
	(N=130)	(N=81)	(N=16)
Sex			
Male	78	49	9
Female	52	32	7
Age (years)			
Mean	78.0	65.6	68.3
Range	26–89	35–84	51-86
Hemodialysis period			
Mean	9.7	11.9	3.5
Range	1–42	1–35	1-11
Primary disease			
Chronic glomerulonephritis (CGN)	62	49	2
Diabetes	32	19	8
Renal sclerosis	15	10	3
Cystic kidney	8	0	0
Ureteral stone	1	0	0
Other	12	3	3
Erythropoiesis-stimulating agent			
Darbepoetin alfa	121	68	16
Epoetin beta pegol	9	11	0
No administration	0	2	0
Iron supplement			
Sodium ferrous citrate	32	22	1
Ferrous fumarate	5	4	0
Saccharated ferric oxide	50	26	11
Sodium ferrous citrate	8	7	0

+ Saccharated ferric oxide			
No administration	35	22	4

Supplementary table 2. Ranges of blood examination dataset

	Dataset	Examination item	Start	End
Hospital S	S_1 (*N*=130)	Hb (g/dl)	10.3±0.8	10.8±0.6
		MCV (fl)	89.6±7.3	92.5±5.3
		TSAT (%)	21.6±11.4	31.7±29.6
		Ferritin (ng/ml)	53.7±50.3	72.7±46.7
	S_2 (*N*=81)	Hb (g/dl)	10.5±0.8	10.9±0.9
		MCV (fl)	92.0±7.0	92.6±6.1
		TSAT (%)	21.7±10.0	26.8±14.1
		Ferritin (ng/ml)	52.6±37.8	61.8±41.3
Hospital K	K_1 (*N*=16)	Hb (g/dl)	11.1±0.7	10.9±0.7
		MCV (fl)	93.8±5.7	95.3±4.5
		TSAT (%)	23.2±10.1	23.4±6.4
		Ferritin (ng/ml)	50.1±31.9	34.8±14.0

Supplementary table 3. Mean ESAs administered dosage

		Darbepoetin alfa	Epoetin beta pegol
Hospital S	S_1 (*N*=130)	20.2±10.1 µg/week<br>(*N*=120)	26.1±8.9 µg/2 weeks<br>(*N*=10)
	S_2 (*N*=81)	18.8±14.1 µg/week<br>(*N*=68)	36.0±15.7 µg/2 weeks<br>(*N*=11)
